# Supplementary material for: Finding defects in glasses through machine learning
Source: Nat Commun. 2023 Jul 15;14:4229. doi: 10.1038/s41467-023-39948-7 (PMC10349890; doi:10.1038/s41467-023-39948-7)
Supplement: Supplementary file 1 — Supplementary Information [file 41467_2023_39948_MOESM1_ESM.pdf]

Supplementary information for Finding defects in glasses through machine learning

Simone Ciarella,[1,*] Dmytro Khomenko,[2,3,*] Ludovic Berthier,[4,5] Felix C. Mocanu,[1] David R. Reichman,[2] Camille Scalliet,[6] and Francesco Zamponi[1]

[1] *Laboratoire de Physique de l'École Normale Supérieure, ENS, Université PSL, CNRS, Sorbonne Université, Université de Paris, 75005 Paris, France*

[2] *Department of Chemistry, Columbia University, 3000 Broadway, New York, NY 10027, USA*

[3] *Dipartimento di Fisica, Sapienza Università di Roma, P.le A. Moro 2, I-00185, Rome, Italy*

[4] *Yusuf Hamied Department of Chemistry, University of Cambridge, Lensfield Road, Cambridge CB2 1EW, United Kingdom*

[5] *Laboratoire Charles Coulomb (L2C), Université de Montpellier, CNRS, 34095 Montpellier, France*

[6] *DAMTP, Centre for Mathematical Sciences, University of Cambridge, Wilberforce Road, Cambridge CB3 0WA, United Kingdom*

SUPPLEMENTARY NOTE 1: WHICH MACHINE LEARNING APPROACH SHOULD WE USE?

A. Comparison between ensembled and non-ensembled models

In the main manuscript we discuss how to transform the problem of computing the quantum splitting of two inherent structures into a supervised learning regression problem. Still, many machine learning models can in principle answer this question. We opted for the AutoGluon library [1] which is based on model ensembling to find an optimal solution. Model ensembling consists in averaging the output of different ML models and create an ensemble-averaged model that outperforms each one of its components. In particular, the AutoGluon library performs model ensembling in the form of stacking (stacking different layers of models that are applied in series) and bagging (training multiple instances of the same model on different subsets of data and then combining their prediction).

In Supplementary Fig. 1(a) we report the most performant ensembling after 20 hours of training, at $T_f = 0.062$, $M = 5$ and $N_{\mathrm{samples}} = 10^4$. The R2-score reported in the figure shows that the most performant model is a bagged ensemble of *CatBoost*, a gradient boosting method [2], which then corresponds to the final *WeightedEnsemble* for this training instance. Notice that in the main paper, we always use the best ensembled model. In addition to *CatBoost*, very good performances are also usually achieved by *LightGBM*, another gradient boosting method [3], and *ExtraTrees*, which is an extremely randomized tree ensemble [4]. Overall, gradient boosting methods typically achieve the highest R2-score. Thus, in the main text when we report results obtained with the 'best models', we refer to ensembles of gradient boosting methods assembled with Autogluon.

We motivate our choice of complex ensemble model constructed with Autogluon by showing that simpler models do not perform at the same level. For the system at $T_f = 0.062$ and in the optimal condition corresponding to $M = 5$ and $N_{\mathrm{samples}} = 7000$ the simplest model to at least capture some correlation was a Multi-layer-perceptron (MLP) with 10 hidden layers of size $1.1 \cdot N_{\mathrm{features}}$, using the same input and output structure as in the main text. A first disadvantage of this approach is that several additional hyperparameters have to be selected, such as learning rate, number and size of hidden layers, activation function, etc. We varied these parameters and report in Supplementary Fig. 1(b) results for the model with the best performance. This optimal MLP was trained until the iteration per epoch was consistently below 10^{-10}, corresponding to ~ 10 hours. Still, the results in Supplementary Fig. 1(b) demonstrate that the MLP performance is much worse than that of the ensemble models used in the main text. We believe that with sufficient training time and optimal hyperparameters, the MLP can perform better. However, our model ensembling approach is much faster and does not require any hyperparameter optimization, so we decided to not invest too many computational resources in the MLP. In particular, we believe that the model in Supplementary Fig. 1(b) is overfocusing on the low quantum splitting pairs, that weight more in the loss function defined in Eq. (3). While in this case the predictions could be scaled by a constant to achieve significant improvement, the problem with such an approach is that it is not reliable, and this scaling constant is not known a priori adding an additional layer of complication. For this reason we prefer to use more complex machine-learning models.

---

* These authors contributed equally.

email: simone.ciarella@ens.fr, dmytro.khomenko@uniroma1.it

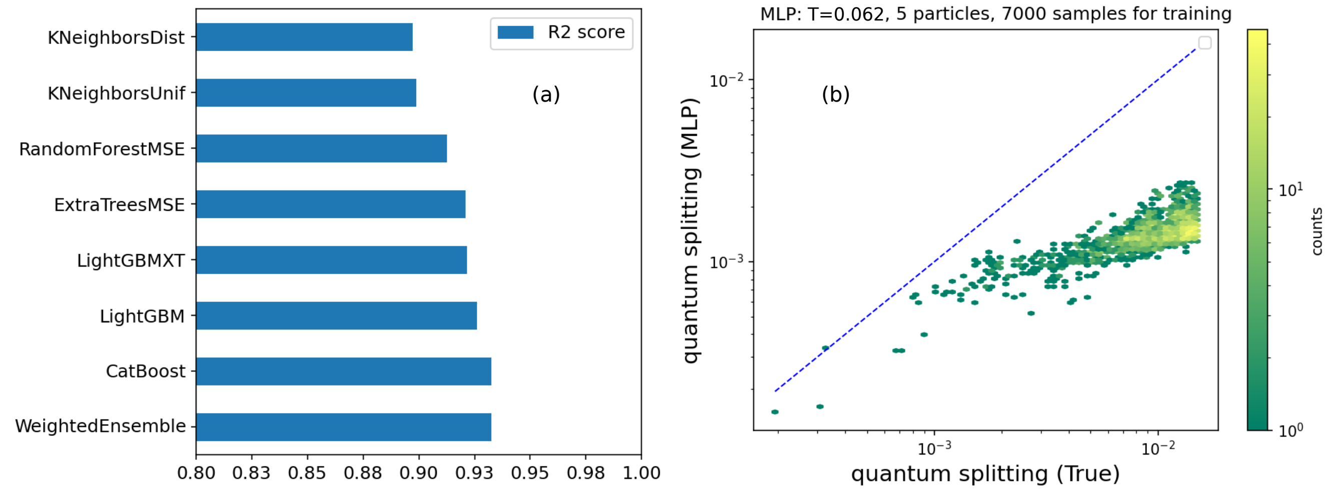

Supplementary Figure 1. Comparison of different ML model ensembles to predict the quantum splitting at $T_f = 0.062$ using $M = 5$ and $N_{\text{samples}} = 7 \cdot 10^4$. (a) R2-score of the test set obtained for an ensemble of models. The full collection was trained for 20 hours of CPU time. In the main manuscript we use the *WeightedEnsemble*. (b) An optimal Neural Network (MLP with 10 hidden layers of size $1.1 \cdot N_{\text{features}}$) does not produce good predictions ($R2 < 0$).

B. Parameters to predict the quantum splitting

A significant advantage of the model ensemble approach is that most of the hyperparameters are automatically optimized by the ensembling. Still, some degrees of freedom have to be fixed. In particular we need to fix the number of particles to be considered (M), how many samples to use (N_{samples}) and for how long to train the ensemble. In the next sections we motivate the choices reported in the main text.

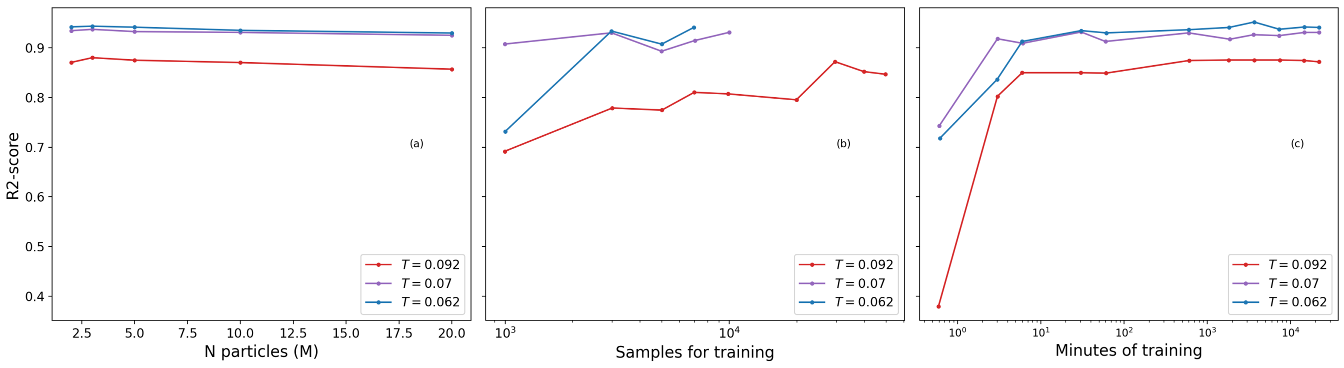

Supplementary Figure 2. Optimizing the Quantum splitting predictor. Effect of (a) the number of particles M, (b) number of samples N_{samples} and (c) training time on performance, while other parameters assume their optimal value. We report the R2-score after training on the data of Ref. [5]. Overall we conclude that optimal performances can be achieved for $M = 3$, $\sim 10^4$ samples in just 10 minutes of training.

- M**: input particles** – In Supplementary Fig. 2(a) we report the effect of M, the number of particles considered in the ML procedure. A large M hinders the performance of the ML model because it has to process a larger input vector. The peak performance is achieved at $M = 3$, which is a relatively low number of particles to define a TLS. This confirms that the participation rate in TLS is low [5].

- N_{samples}**: number of samples** – To evaluate the optimal value of N_{samples} we report in Supplementary Fig. 2(b) the variation of the R2-score upon using more samples for training. Notice that we keep the other parameters in the optimal condition for each temperature. The shape of the curves let us conclude that a good number of training samples is $7000, 10000, 30000$ over a total of $14202, 23535, 117370$ for $T_f = 0.062, 0.07, 0.092$ respectively.

Notice that the results depend on the specific quality of the samples provided for training. It is possible to achieve better predictions by selecting better initial samples, more uniformly distributed, which are different from the random choice we make for simplicity. Still, the values reported consistently produce good results, independently from the initial sample composition.

- **Training time** – In Supplementary Fig. 2(c) we report the effect of the training time on performance. The R2-score approaches the plateau in 10 minutes of training on a single CPU and reaches the final value after 10^3 minutes (~ 16 hours). Overall, we find that in iterative training, where we retrain the model several times, a good balance between performance and speed can be reached by training for ~ 10 minutes. Instead, in a more classic single training approach we suggest to train for > 10^3 minutes. Performing the training in parallel can further reduce the training time.

C. Parameters to identify double wells

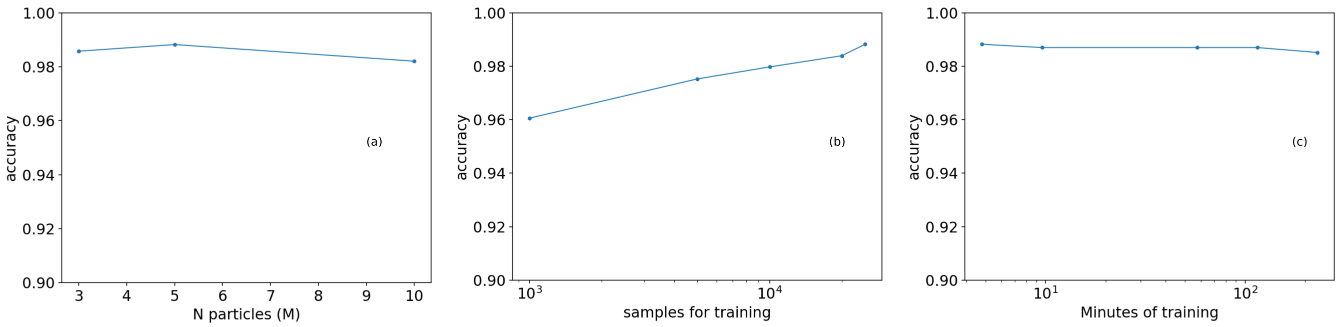

Supplementary Figure 3. Accuracy of the double well classifier as a function of (a) M, (b) training time, and (c) number of samples. We report the accuracy after training at the lowest temperature $T_f = 0.062$ using the data from Ref. [5].

The ML model has to process a large number of unfiltered minima obtained during the exploration simulation. While we can anticipate that only a small fraction of inherent structure pairs will be a TLS, we also know that the minimum energy path connecting any pair of IS will not form a DW and as it is likely to contain intermediate minima. To exclude those non-DW pairs, we train a classifier using as input the same quantities that we measure to predict the QS, as described in the main text. In Supplementary Fig. 3 we report the results of a hyperparameter scan at $T_f = 0.062$. These results are similar to those obtained for the QS prediction suggesting that at $T_f = 0.062$ we can use (a) $M = 3$, (b) 10^2s of training and (c) ~ 10^4 samples in order to achieve > 95% accuracy, measured over a validation set. We conclude that we can use the same hyperparameters for the DW classifier and the QS predictor.

SUPPLEMENTARY NOTE 2: FEATURES

D. Removing irrelevant features

After finding the optimal parameters to train the ML model, we can extract the specific effect and overall importance of each input parameter, as well as the score drop when each of them is removed. Since each additional feature corresponds to additional computational time and memory, we investigate which features are crucial, and which ones can be excluded to make the ML pipeline faster and more efficient without affecting performance.

In Supplementary Fig. 4(a) we report the feature importance (in log scale) after a single iteration of training containing all the data from Ref. [5]. Different colors refer to glasses prepared at different temperatures. Independently from the temperature, the most important features are the energy difference ΔE between the two IS, followed by the total displacement of particles $\sum_i \Delta\vec{r}_i$. The third most important information is the positions of the particles that displaced the most $\sum_i |\vec{r}_1 - \vec{r}_i|$. After them, we find that the arrangement of the first shell of neighbors represented by the q parameters and the particle sizes σ_i are insignificant (and so is their variation between the two IS of the pair $\Delta\cdot$ reported in Supplementary Fig. 4), since their importance is more than two orders of magnitude smaller than the one of the energy and displacements. Since the q parameters are also the slowest to compute, we decided to not include them in the final pipeline reported in the main manuscript.

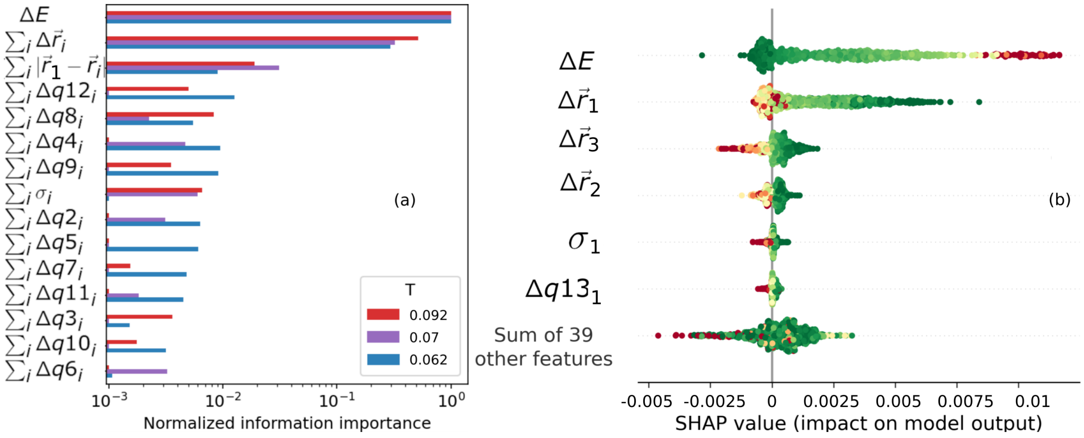

Supplementary Figure 4. Importance of different features on the quantum splitting predictor. (a) Performance drop when each specific feature is removed, normalized to the first most important. Different colors correspond to different glass preparation temperatures.(b) Shapley values calculated at $T_f = 0.062$. In general, at any temperature the most important feature is the energy difference (ΔE) of the two IS, which is followed by the value of the displacement of the M particles. The Shapley values on the right also report the effect of the specific features: the splitting ΔE has to be small (green) in order to predict low QS (i.e. negative SHAP), while instead the displacements have to be large (red) in order to point towards low QS.

Next, to quantify the role of those inputs, we calculate their Shapley values [6] shown in Supplementary Fig. 4(b) for $T_f = 0.062$. The color codes for the value taken by the specific feature (red when the value is high, green when low) and reports its scaled impact on the model output. The x axis reports the Shapley value, where SHAP>0 implies that the specific feature is pushing towards predicting a high QS, while SHAP<0 indicates that the feature promotes low QS values. While no input feature alone can predict a TLS (because |SHAP| is small) there are some visible trends: (i) the energy asymmetry is the most important feature and should be small in order to predict a low QS, (ii) the particle displacements have to be larger than a threshold in order to predict low QS.

According to these results we rationalize the ML prediction. The energy difference between two IS is the main predictor for the quantum splitting, which is never too large for DW, then the displacements are necessary to understand if the two IS are similar and what their stability is (see the temperature classification section in the SI). The displacement nucleus size complements the information contained in the displacements and gives local information about the participation ratio. Finally, all the other features do not provide any improvement. Since the bond order parameters are computationally expensive to calculate, we do not include them in the final ML approach we propose in the main text. The performance reported in the main text confirms that our choice is justified.

E. Reducing the number of features

We justify the choice of features discussed in sec. IVa and Fig. 2 by presenting the performance of our ML model as a function of feature number. In Supplementary Tab. I we rank the features according to their Shapley values (see Fig. 6). We report (blue line) in Supplementary Fig. 5 the accuracy of the DW classifier (a) and the QS predictor (b) as a function of number of features, following the Shapley ranking of Supplementary Tab. I. The DW classifier reaches its best accuracy with six features. In the initial study Ref. [5, 7], the matrix $\mathbf{T}$ was the only information used to analyze the pair of IS. Here, the classifier already reaches $\sim 90\%$ accuracy using the transition matrix only (two features). We add (orange line) the performances when we exclude $\Delta E, T_{\alpha\beta}$ and $T_{\beta\alpha}$, which are the most important features overall. Surprisingly, the DW classifier still reaches its maximum accuracy with two features ($\Delta\vec{r}_3$ and $|\vec{r}_1 - \vec{r}_3|$) as highlighted in the inset. The QS predictor shows instead very good predictions even using a single feature (ΔE in the blue curve, or $\Delta\vec{r}_1$ in the orange).

Shapley ranking	1st	2nd	3rd	4th	5th	6th	7th	8th	9th	10th
**DW classification**	$T_{\alpha\beta}$	$T_{\beta\alpha}$	$\Delta\vec{r}_3$	$\lvert\vec{r}_1 - \vec{r}_3\rvert$	$\lvert\vec{r}_1 - \vec{r}_2\rvert$	d	$\Delta\vec{r}_1$	ΔE	PR	$\Delta\vec{r}_2$
**QS prediction**	ΔE	$\Delta\vec{r}_1$	d	$\Delta\vec{r}_2$	$\lvert\vec{r}_1 - \vec{r}_2\rvert$	$\lvert\vec{r}_1 - \vec{r}_3\rvert$	$\Delta\vec{r}_3$	$T_{\alpha\beta}$	$T_{\beta\alpha}$	PR

Table I. Ranking the features used in the main manuscript by their Shapley values.

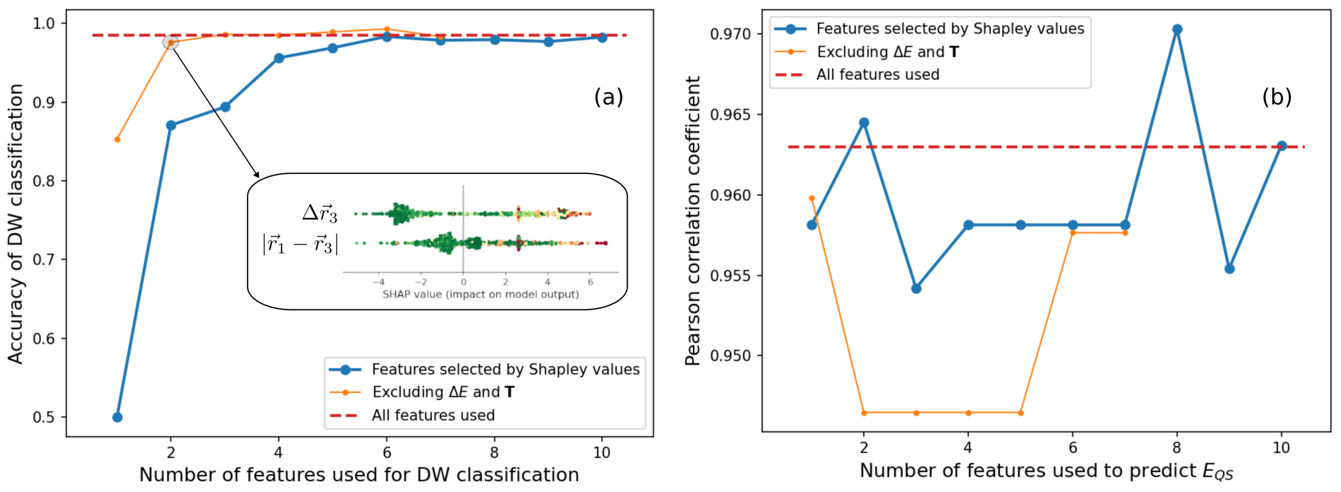

Supplementary Figure 5. Effect of the number of features. Accuracy of the DW classifier (a) and Pearson correlation score of the QS predictor (b), as a function of the number of features used. Along the blue curves the features are ranked by their importance using their Shapley values, so the first features to be used are the most important. For the orange curve we exclude ΔE, $T_{\alpha\beta}$ and $T_{\beta\alpha}$ to evaluate the performances of the ML model without the features with the best Shapley values.

SUPPLEMENTARY NOTE 3: ITERATIVE TRAINING

The standard supervised learning approach consists in collecting a significant amount of data, then using them to train the ML model and finally use the model predictions. While overall very powerful, this scheme is not optimal when the goal of the model is to drive the exploration in a space much larger than the available data. This is the case of our main study where we employ the ML model to identify IS pairs with low QS. In order to make our approach more efficient and generalizable we developed the *iterative training* procedure.

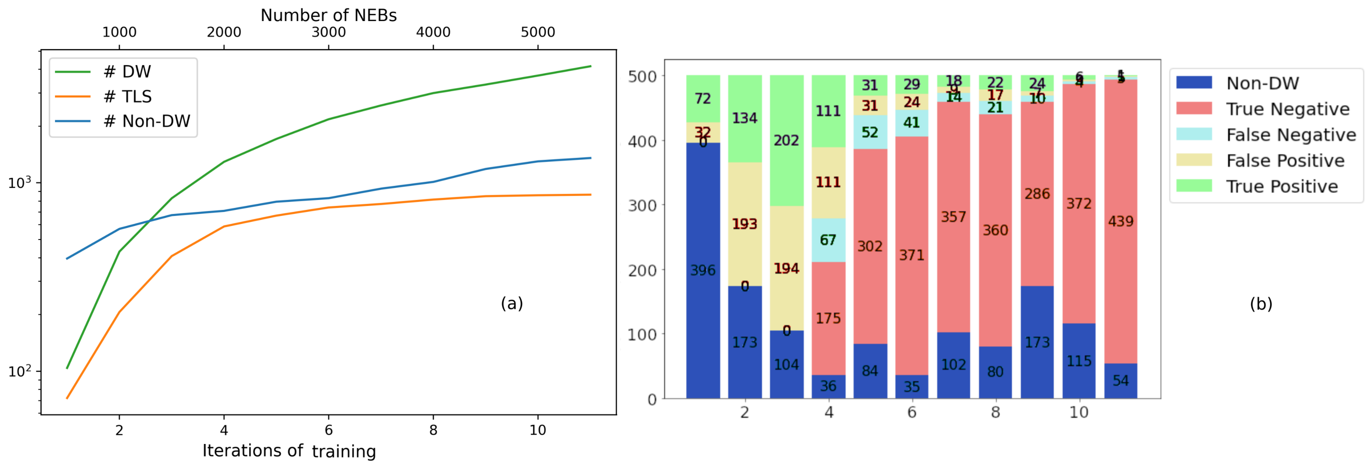

Supplementary Figure 6. Performance of the iterative training procedure at $T_f = 0.062$ for the data collected in the main manuscript. Starting from $K_0 = 5000$ samples we perform iterations of $K_i = 500$ predictions for 11 iterations. In (a) we report the cumulative number of double wells (DW), two-level systems (TLS) and non double wells (Non-DW). In (b) we break down this result by color coding the confusion matrix of the ML model at each iteration of iterative training.

We start the iterative training procedure from a sample of K_0 pairs for which we calculate the QS. We empirically find that $K_0 \sim 5000$ achieves a good balance between precision and time. This sample has to be balanced between DW and non-DW, but there is no need to include TLS in the initial sample. It is possible to start from a smaller K_0, at the cost of a performance drop in the first few iterations of training. From the initial sample we perform a first training that takes 10 minutes for the DW classifier and 10 minutes for the QS predictor. We then use the model to predict the $K_i = 500$ IS pairs with the lowest QS. We report the cumulative number of TLS, DW, and non-DW at

each iteration in Supplementary Fig. 6(a). From Supplementary Fig. 6(b) we see that during the first iteration most of the $K_i = 500$ best candidates are actually non-DW, so the model is performing poorly. This is the reason why we suggest to perform only $K_i = 500$ NEBs for each iterations, otherwise the first iteration would lead to wasting time to perform calculations over non-DW. On the other hand, in the first iteration 72 TLS are already found by running 500 NEBs. This is to be compared with Ref. [5] in which 61 TLS were found by running > 14000 NEBs. After the first retraining (during iteration 2) the performance of the ML model is already excellent and less than 30% of the 500 best pairs are non-DW, while 134 are newly found TLS.

We also used our iterative training to reprocess the data of Ref. [5], and we report the results in Table 1 (main text). We show in Supplementary Fig. 7 a detailed analysis of the procedure at $T_f = 0.062$. While the standard approach was able to identify 61 TLS running > 14000 NEB+Schrödinger calculations, iterative training finds 156 TLS, by running only 2500 NEBs. This confirms that more than half of the total TLS were hidden among the pairs discarded by Ref. [5]. In details, Supplementary Fig. 7(a) shows the cumulative number of DW and TLS that we find, while Supplementary Fig. 7(b) reports the confusion matrix for the different steps of iterative training.

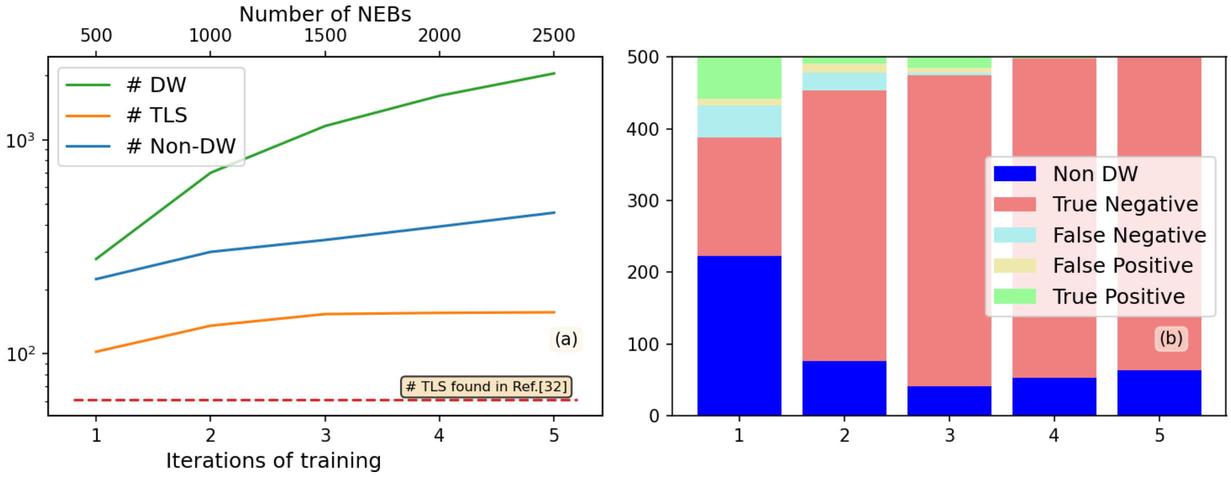

Supplementary Figure 7. Reprocessing the data of Ref. [5] at $T_f = 0.062$ with iterative training. In (a) we report the cumulative number of double wells (DW), two-level systems (TLS) and non double wells (Non-DW). While Ref. [5] (red dashed line) identified 61 TLS running > 14000 NEBs, iterative training finds 156 TLS from 2500 NEBs. In (b) we report the confusion matrix of the 5 steps of iterative training that we run.

Overall, these results demonstrate that the ML approach is not only faster than a manual filtering rule based on the transition matrix, but also much more effective. The pool of IS pairs excluded from the analysis in the original approach effectively contains a significant number of TLS. In conclusion, a ML driven exploration is not only more efficient than manual filtering, but also necessary to capture the correct statistics.

SUPPLEMENTARY NOTE 4: TEMPERATURE AND STABILITY

Our ML approach is able to predict the quantum splitting of a pair of IS from static information. It is known that TLS have different features depending on their preparation temperature, or glass stability [5]. Here we address the following questions: (i) how difficult is it for a machine to distinguish data corresponding to different temperatures, (ii) what are the most important features for this task, and (iii) does our ML approach learn temperature-independent features?

F. Temperature classification

To understand how TLS and IS features evolve with temperature, we trained a multi-layer-perceptron (MLP) to classify the temperature corresponding to a specific pair. We find that it is possible to rapidly train a classifier reaching accuracy > 95%. Depending on the value of M, the input layer is composed by N_{features} neurons, where the features are explained in the main text. After the input layer, there are n hidden fully connected layers, all of the same size s. The activation function for each neuron-neuron connection is a ReLu function. The last layer is composed of 3 neurons that represent the probability that a given input belongs to one of the three classes: $T_f = 0.062$ or $T_f = 0.07$

or $T_f = 0.092$. The performance of the MLP are evaluated by measuring the accuracy, which is the percentage of the corrected predictions. In Supplementary Fig. 8(a) we report the effect of increasing the size of the hidden layers, while in Supplementary Fig. 8(b) we report the effect of a larger number of hidden layers.

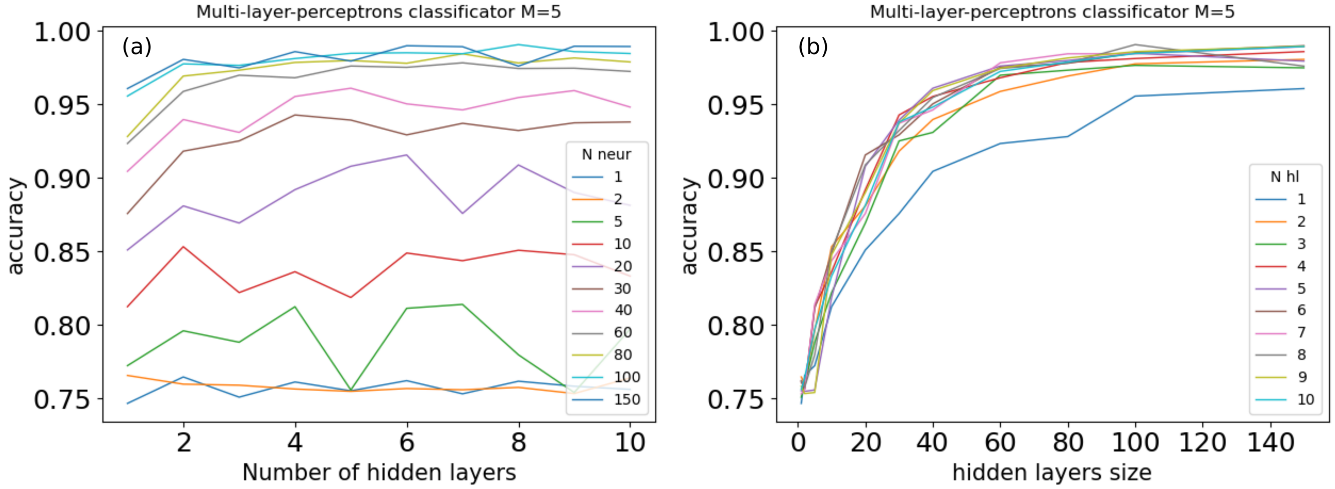

Supplementary Figure 8. Temperature classifier accuracy as a function of number of hidden layers (a) and their size (b). We report results for $M = 5$. Results suggest that already with 2 hidden layers of 60 neurons it is possible to achieve an accuracy above 95%.

Our results show that a MLP with 2 hidden layers of 60 neurons has a 95% accuracy after less than 1h of supervised training. The answer to question (i), is that one can identify the temperature at which a pair of IS was obtained.

G. Static signatures of glass stability

We answer question (ii) by measuring the Shapley (SHAP) values from the temperature classifier. In Supplementary Fig. 9 (a) we report the SHAP value of the most important input features for the MLP prediction that considers only the $M = 5$ particles that displaced the most. The smallest ($i = 5$) and the largest ($i = 1$) particle displacements emerge as the most important input, noted $\Delta\vec{r}_5$ and $\Delta\vec{r}_1$, respectively. Their average effect on the model prediction is shown in Supplementary Fig.9(b,c). The particle that displaced the most ($i = 1$) shows a large displacement at low preparation temperature, while the particle that displaced the least ($i = M$) exhibits a large displacement at high temperature. Our results suggest that higher temperatures are characterized by more collective rearrangements, leading to large $\Delta\vec{r}_5$. In glasses prepared at low temperature instead, transitions are characterized by a particle displacing significantly more than the others.

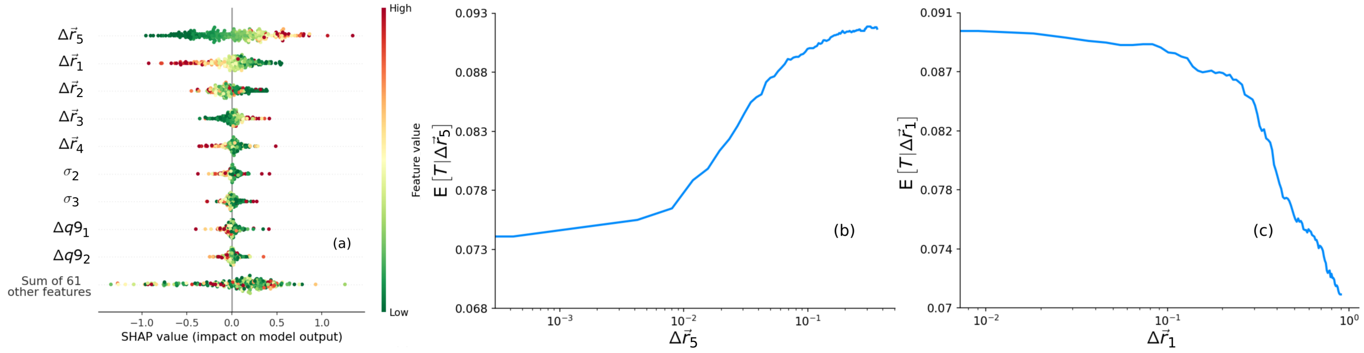

Supplementary Figure 9. Microscopic differences originating from different temperature preparation/glass stability, quantified using the Shapley values for the temperature classifier. (a) Summary of the SHAP values for the 9 most important input features, measured for a subset of 1000 pairs processed by the T-classifier. Predicted glass preparation temperature as a function of (b) $\Delta\vec{r}_5$, and (c) $\Delta\vec{r}_1$, with all other inputs taking their average value.

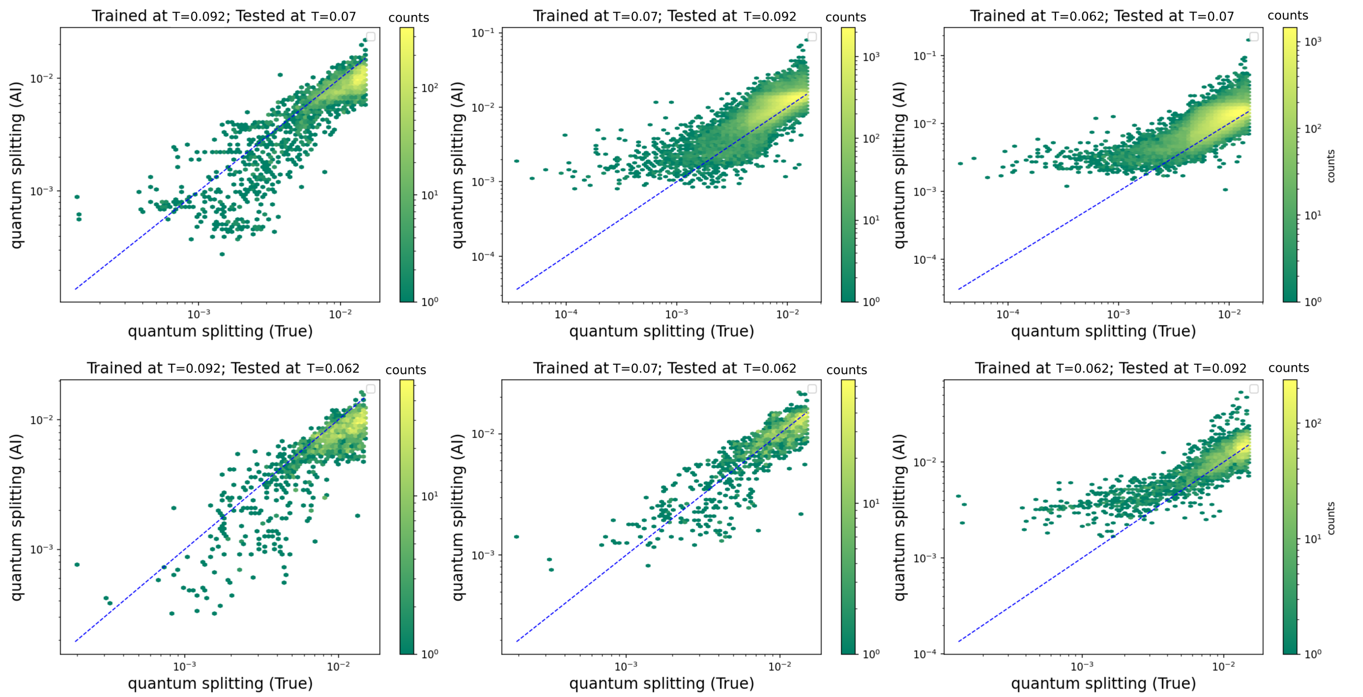

Supplementary Figure 10. Transferability and crossvalidation of the machine learning model. Exact quantum splitting against ML pre-diction. The quantum splitting predictor is trained at T_{train} and deployed at $T_{\text{prediction}} \neq T_{\text{train}}$. From left to right: $T_{\text{train}} = 0.092, 0.07, 0.062$. From top to bottom, decreasing $T_{\text{prediction}}$. Performances are not on par with Fig. 3, but good in particular when $T_{\text{train}} > T_{\text{predict}}$, which is of practical interest.

H. Transferability and crossvalidation

We address question (iii) by testing how our model performs when trained at T_{train} and deployed to predict the quantum splitting of pairs at $T_{\text{predict}} \neq T_{\text{train}}$. In Fig. 10 each column corresponds to a training temperature: $T_{\text{train}} = 0.092$ (left), 0.07 (middle) and 0.062 (right). Then, computed quantum splittings are compared with ML prediction made on samples obtained at T_{predict}, decreasing from top to bottom. We see in Supplementary Fig. 10 that the predictive power of a model trained at a different temperature is slightly lower compared to the model trained at the same temperature (Fig. 3 of main). Still, the transferability of the model is good, especially when the model is trained at $T_{\text{train}} > T_{\text{predict}}$. This situation is the most useful, since it is easier to obtain data at higher T. We conclude that if not enough data is available at low temperature, it is possible to rely on model transferability. This relies on the fact that TLS share some general temperature-independent features.

In summary, we have seen that IS and TLS have different microscopic features when generated from different stabilities. It is then optimal to train the ML model at a fixed temperature only. If no easier way to measure T/stability are available, it is possible to use ML to classify the stability of each IS by adding another block to the workflow, as reported in the main manuscript. Alternatively, at the cost of a finite accuracy drop it is even possible to transfer the model predictions at different temperatures and thus train where data collection is fast and easy and apply it where it is not.

[1] N. Erickson, J. Mueller, A. Shirkov, H. Zhang, P. Larroy, M. Li, and A. Smola, Autogluon-tabular: Robust and accurate automl for structured data, arXiv preprint arXiv:2003.06505 (2020).

[2] L. Prokhorenkova, G. Gusev, A. Vorobev, A. V. Dorogush, and A. Gulin, Catboost: unbiased boosting with categorical features, Advances in neural information processing systems **31** (2018).

[3] H. Zhang, S. Si, and C.-J. Hsieh, Gpu-acceleration for large-scale tree boosting, arXiv preprint arXiv:1706.08359 (2017).

[4] P. Geurts, D. Ernst, and L. Wehenkel, Extremely randomized trees, Machine Learning **63**, 3 (2006).

[5] D. Khomenko, C. Scalliet, L. Berthier, D. R. Reichman, and F. Zamponi, Depletion of two-level systems in ultrastable computer-generated glasses, Physical Review Letters **124**, 225901 (2020).

[6] S. M. Lundberg, G. Erion, H. Chen, A. DeGrave, J. M. Prutkin, B. Nair, R. Katz, J. Himmelfarb, N. Bansal, and S.-I. Lee, From local explanations to global understanding with explainable ai for trees, Nature Machine Intelligence **2**, 56 (2020).
[7] F. C. Mocanu, L. Berthier, S. Ciarella, D. Khomenko, D. R. Reichman, C. Scalliet, and F. Zamponi, Microscopic observation of two-level systems in a metallic glass model, arXiv preprint arXiv:2209.09579 (2022).
